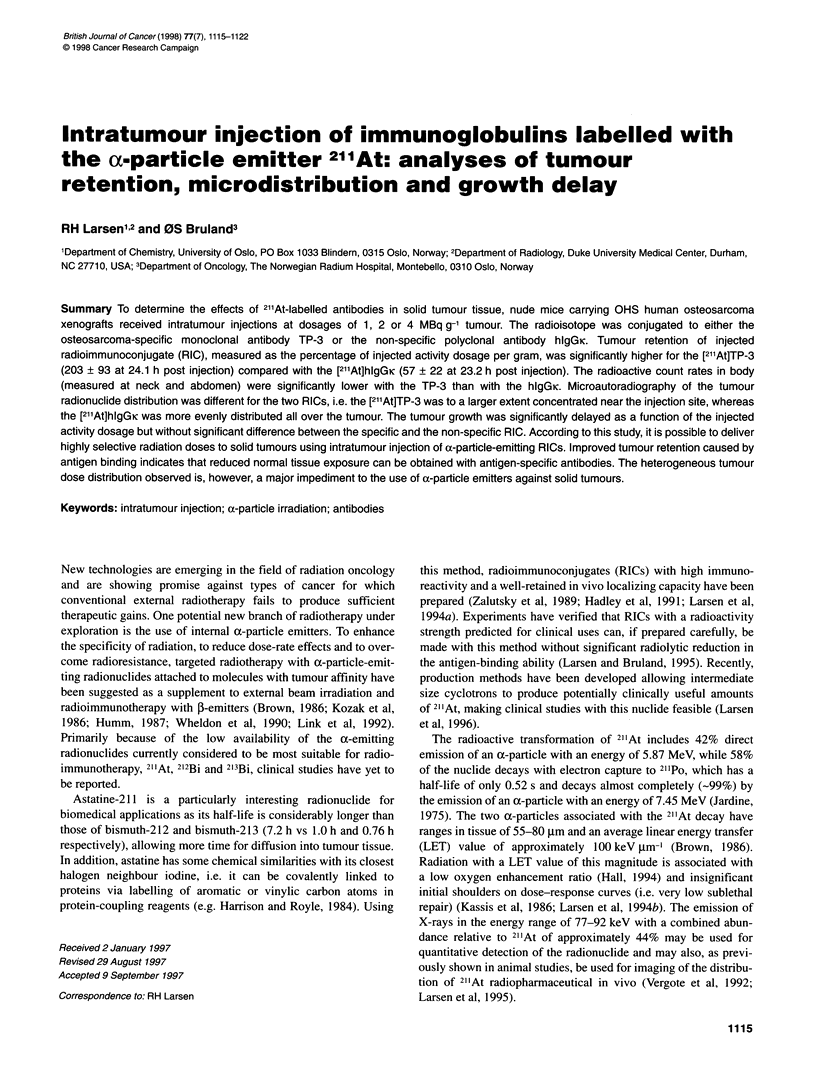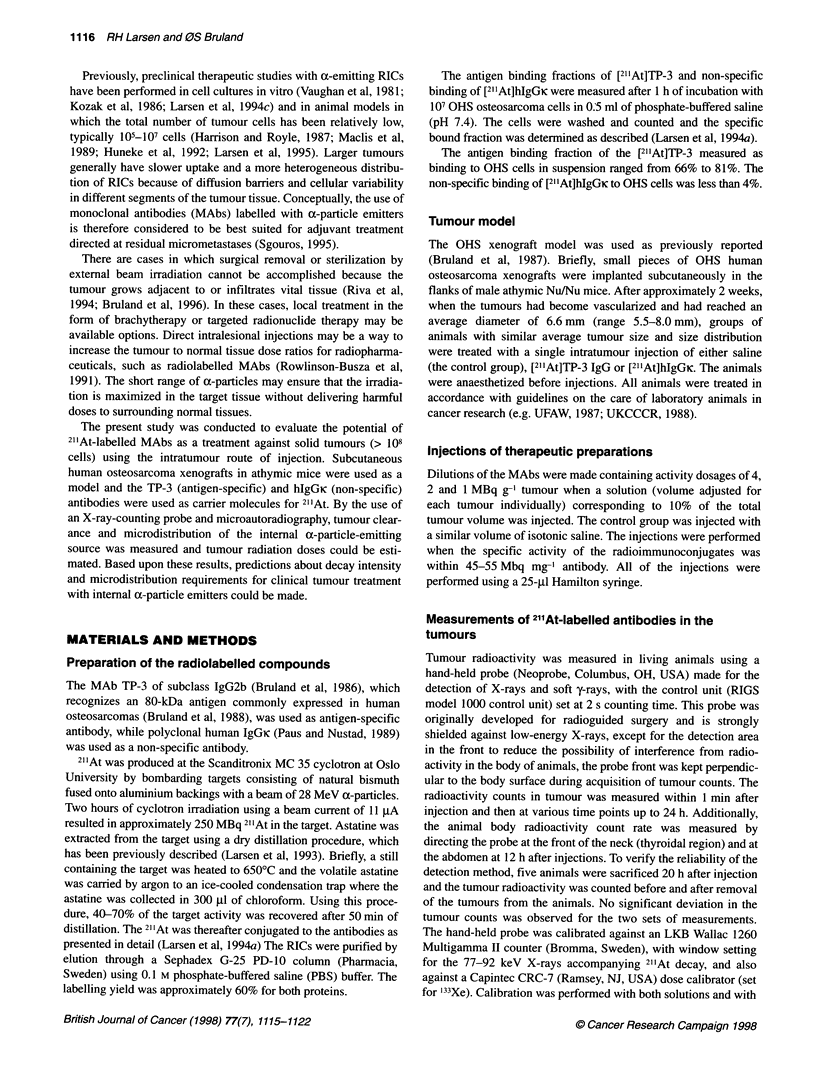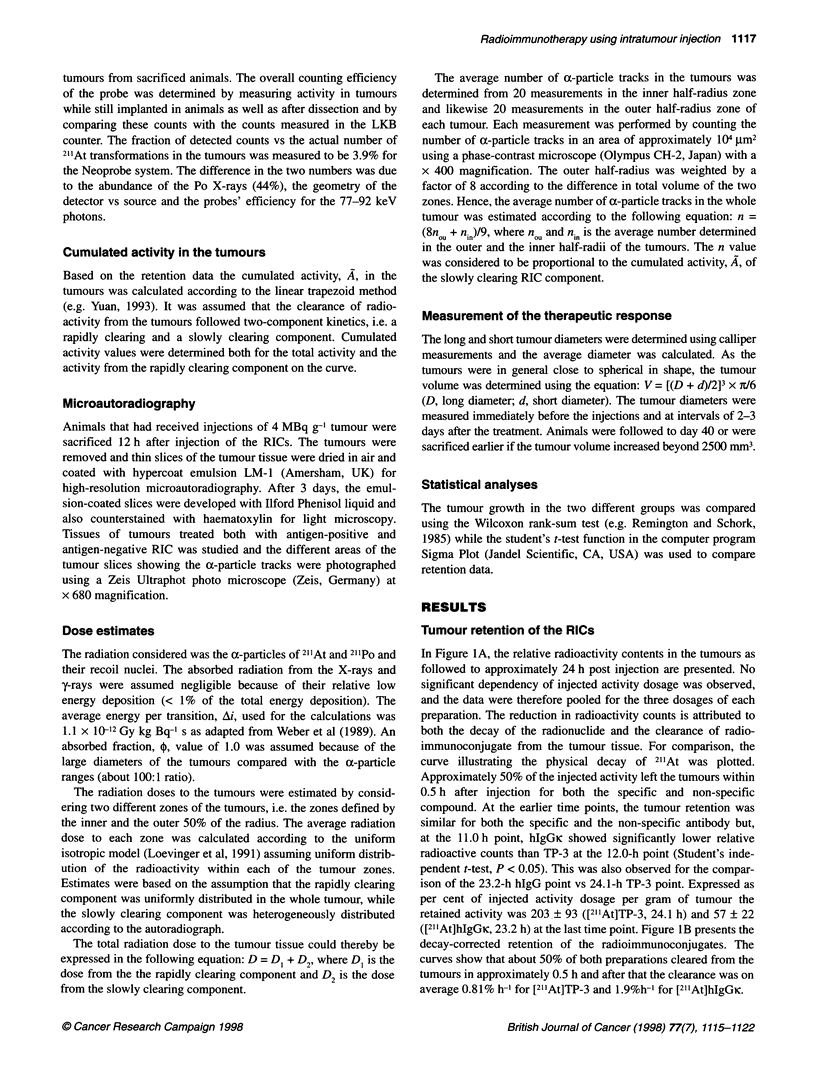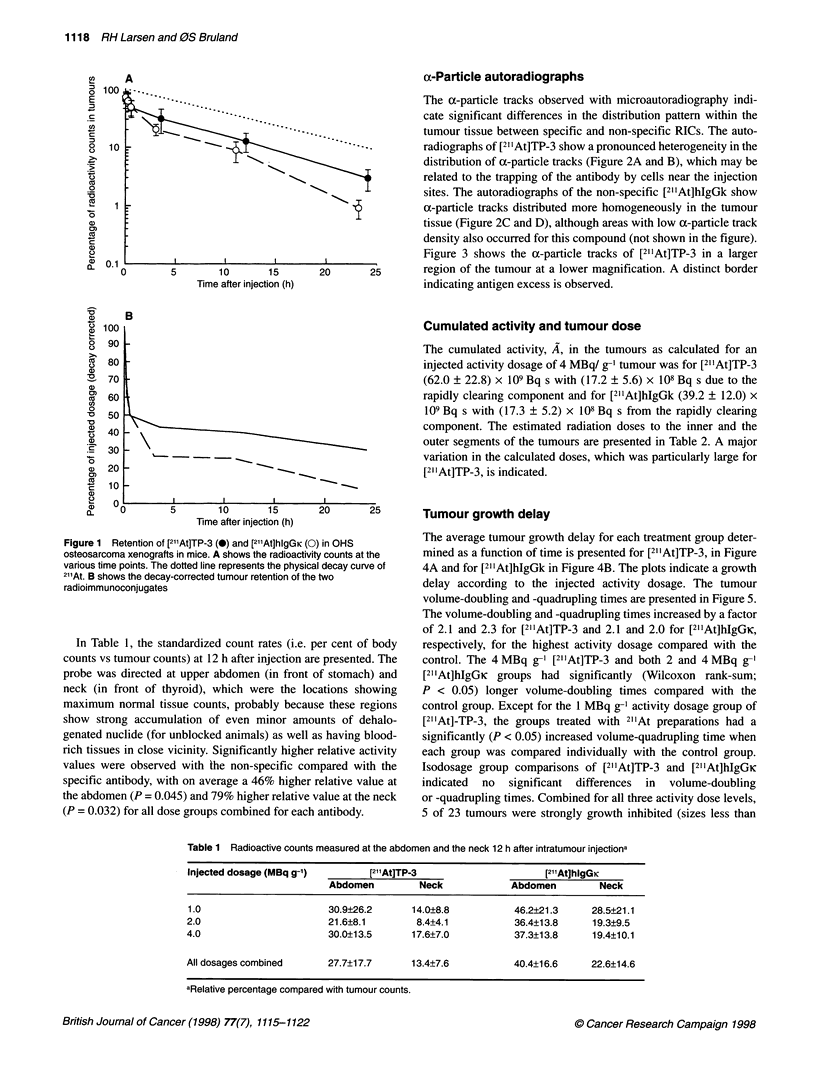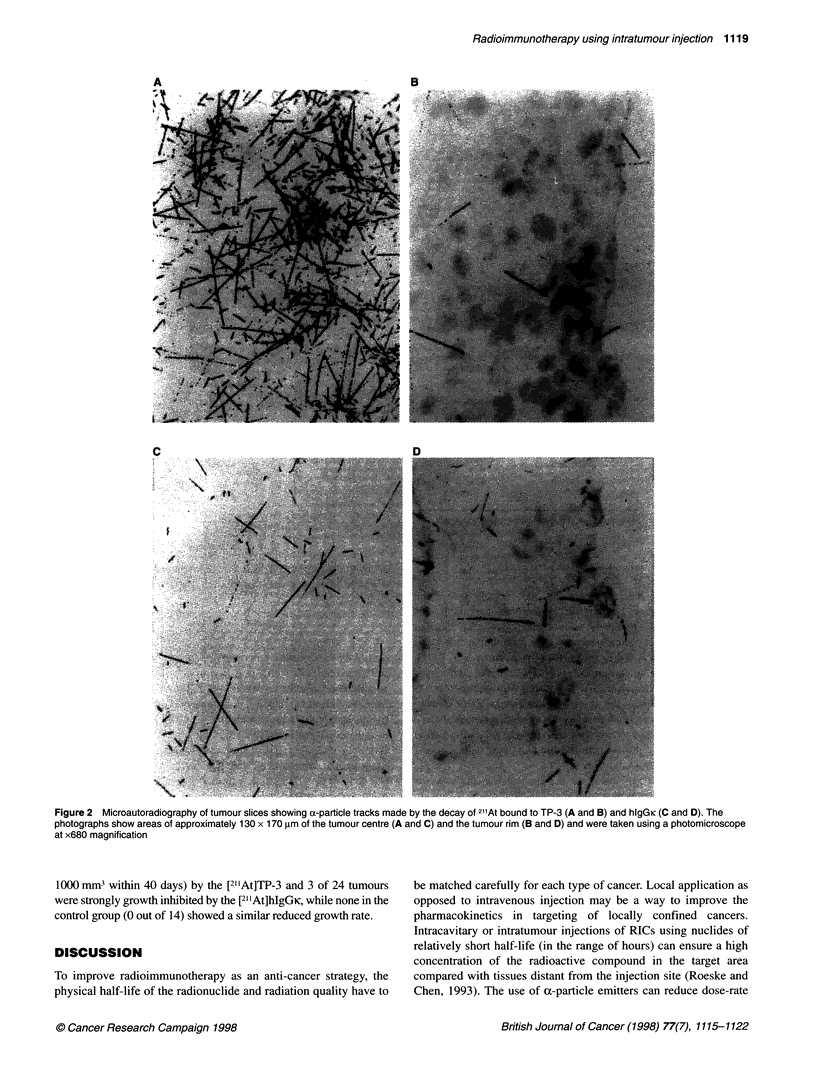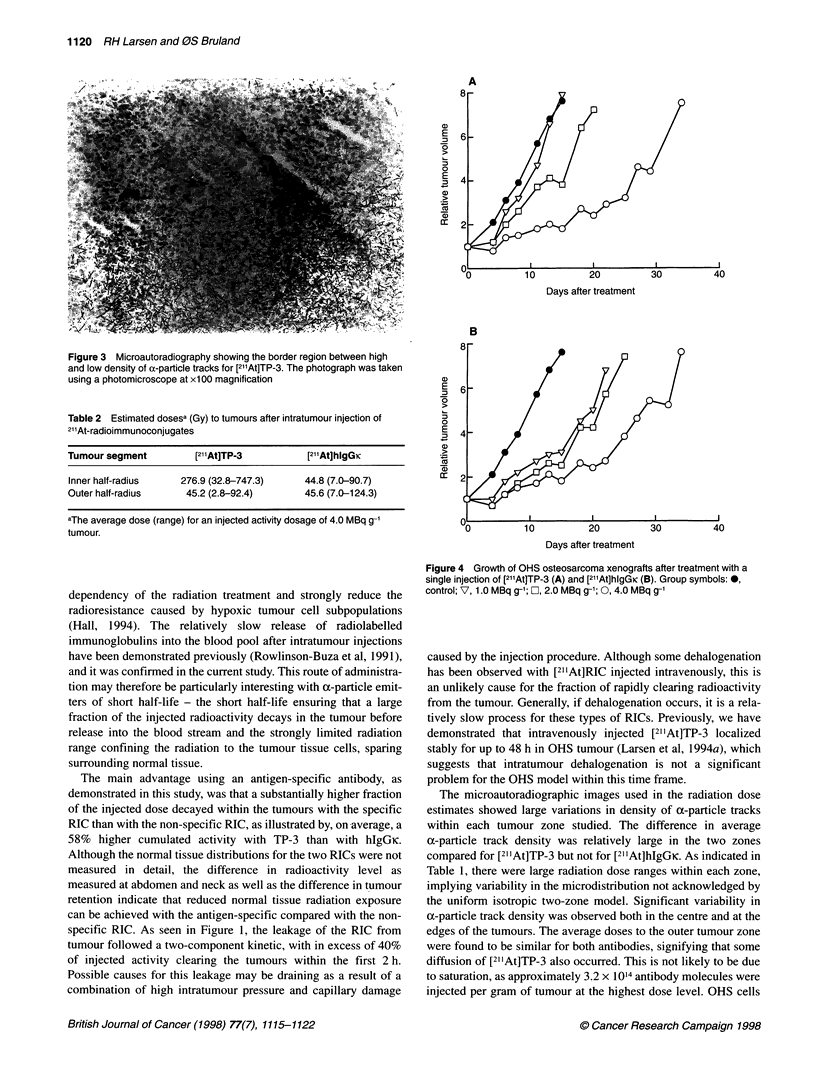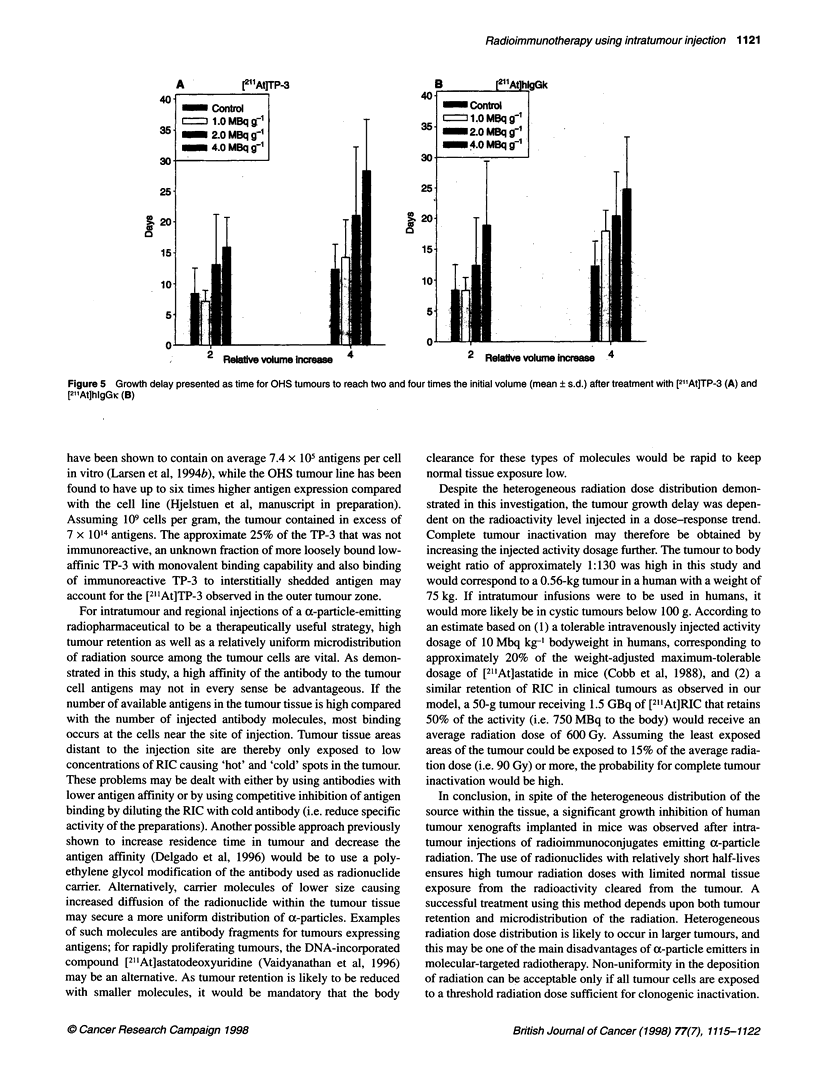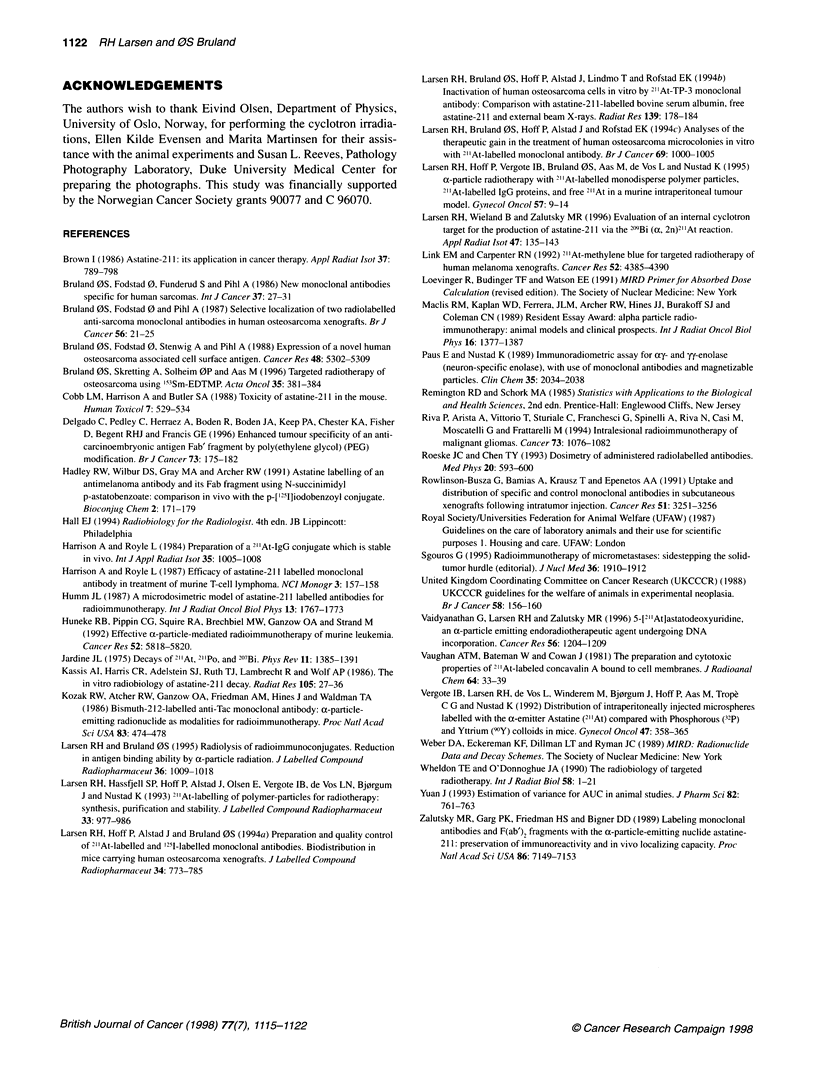# Intratumour injection of immunoglobulins labelled with the alpha-particle emitter 211At: analyses of tumour retention, microdistribution and growth delay.

**DOI:** 10.1038/bjc.1998.185

**Published:** 1998-04

**Authors:** R. H. Larsen, O. S. Bruland

**Affiliations:** Department of Chemistry, University of Oslo, Blindern, Norway.

## Abstract

**Images:**


British Journal of Cancer (1998) 77(7), 1115–1122
© 1998 Cancer Research Campaign

### Intratumour injection of immunoglobulins labelled with the α-particle emitter $^{211}$At: analyses of tumour retention, microdistribution and growth delay

**RH Larsen[1,2] and ØS Bruland[3]**

[1]Department of Chemistry, University of Oslo, PO Box 1033 Blindern, 0315 Oslo, Norway; [2]Department of Radiology, Duke University Medical Center, Durham, NC 27710, USA; [3]Department of Oncology, The Norwegian Radium Hospital, Montebello, 0310 Oslo, Norway

**Summary** To determine the effects of $^{211}$At-labelled antibodies in solid tumour tissue, nude mice carrying OHS human osteosarcoma xenografts received intratumour injections at dosages of 1, 2 or 4 MBq g$^{-1}$ tumour. The radioisotope was conjugated to either the osteosarcoma-specific monoclonal antibody TP-3 or the non-specific polyclonal antibody hIgGκ. Tumour retention of injected radioimmunoconjugate (RIC), measured as the percentage of injected activity dosage per gram, was significantly higher for the [$^{211}$At]TP-3 (203 ± 93 at 24.1 h post injection) compared with the [$^{211}$At]hIgGκ (57 ± 22 at 23.2 h post injection). The radioactive count rates in body (measured at neck and abdomen) were significantly lower with the TP-3 than with the hIgGκ. Microautoradiography of the tumour radionuclide distribution was different for the two RICs, i.e. the [$^{211}$At]TP-3 was to a larger extent concentrated near the injection site, whereas the [$^{211}$At]hIgGκ was more evenly distributed all over the tumour. The tumour growth was significantly delayed as a function of the injected activity dosage but without significant difference between the specific and the non-specific RIC. According to this study, it is possible to deliver highly selective radiation doses to solid tumours using intratumour injection of α-particle-emitting RICs. Improved tumour retention caused by antigen binding indicates that reduced normal tissue exposure can be obtained with antigen-specific antibodies. The heterogeneous tumour dose distribution observed is, however, a major impediment to the use of α-particle emitters against solid tumours.

**Keywords:** intratumour injection; α-particle irradiation; antibodies

New technologies are emerging in the field of radiation oncology and are showing promise against types of cancer for which conventional external radiotherapy fails to produce sufficient therapeutic gains. One potential new branch of radiotherapy under exploration is the use of internal α-particle emitters. To enhance the specificity of radiation, to reduce dose-rate effects and to overcome radioresistance, targeted radiotherapy with α-particle-emitting radionuclides attached to molecules with tumour affinity have been suggested as a supplement to external beam irradiation and radioimmunotherapy with β-emitters (Brown, 1986; Kozak et al, 1986; Humm, 1987; Wheldon et al, 1990; Link et al, 1992). Primarily because of the low availability of the α-emitting radionuclides currently considered to be most suitable for radioimmunotherapy, $^{211}$At, $^{212}$Bi and $^{213}$Bi, clinical studies have yet to be reported.

Astatine-211 is a particularly interesting radionuclide for biomedical applications as its half-life is considerably longer than those of bismuth-212 and bismuth-213 (7.2 h vs 1.0 h and 0.76 h respectively), allowing more time for diffusion into tumour tissue. In addition, astatine has some chemical similarities with its closest halogen neighbour iodine, i.e. it can be covalently linked to proteins via labelling of aromatic or vinylic carbon atoms in protein-coupling reagents (e.g. Harrison and Royle, 1984). Using this method, radioimmunoconjugates (RICs) with high immunoreactivity and a well-retained in vivo localizing capacity have been prepared (Zalutsky et al, 1989; Hadley et al, 1991; Larsen et al, 1994*a*). Experiments have verified that RICs with a radioactivity strength predicted for clinical uses can, if prepared carefully, be made with this method without significant radiolytic reduction in the antigen-binding ability (Larsen and Bruland, 1995). Recently, production methods have been developed allowing intermediate size cyclotrons to produce potentially clinically useful amounts of $^{211}$At, making clinical studies with this nuclide feasible (Larsen et al, 1996).

The radioactive transformation of $^{211}$At includes 42% direct emission of an α-particle with an energy of 5.87 MeV, while 58% of the nuclide decays with electron capture to $^{211}$Po, which has a half-life of only 0.52 s and decays almost completely (~99%) by the emission of an α-particle with an energy of 7.45 MeV (Jardine, 1975). The two α-particles associated with the $^{211}$At decay have ranges in tissue of 55–80 μm and an average linear energy transfer (LET) value of approximately 100 keV μm$^{-1}$ (Brown, 1986). Radiation with a LET value of this magnitude is associated with a low oxygen enhancement ratio (Hall, 1994) and insignificant initial shoulders on dose–response curves (i.e. very low sublethal repair) (Kassis et al, 1986; Larsen et al, 1994*b*). The emission of X-rays in the energy range of 77–92 keV with a combined abundance relative to $^{211}$At of approximately 44% may be used for quantitative detection of the radionuclide and may also, as previously shown in animal studies, be used for imaging of the distribution of $^{211}$At radiopharmaceutical in vivo (Vergote et al, 1992; Larsen et al, 1995).

*Received 2 January 1997*
*Revised 29 August 1997*
*Accepted 9 September 1997*

*Correspondence to:* RH Larsen

Previously, preclinical therapeutic studies with α-emitting RICs have been performed in cell cultures in vitro (Vaughan et al, 1981; Kozak et al, 1986; Larsen et al, 1994*c*) and in animal models in which the total number of tumour cells has been relatively low, typically $10^5$–$10^7$ cells (Harrison and Royle, 1987; Maclis et al, 1989; Huneke et al, 1992; Larsen et al, 1995). Larger tumours generally have slower uptake and a more heterogeneous distribution of RICs because of diffusion barriers and cellular variability in different segments of the tumour tissue. Conceptually, the use of monoclonal antibodies (MAbs) labelled with α-particle emitters is therefore considered to be best suited for adjuvant treatment directed at residual micrometastases (Sgouros, 1995).

There are cases in which surgical removal or sterilization by external beam irradiation cannot be accomplished because the tumour grows adjacent to or infiltrates vital tissue (Riva et al, 1994; Bruland et al, 1996). In these cases, local treatment in the form of brachytherapy or targeted radionuclide therapy may be available options. Direct intralesional injections may be a way to increase the tumour to normal tissue dose ratios for radiopharmaceuticals, such as radiolabelled MAbs (Rowlinson-Busza et al, 1991). The short range of α-particles may ensure that the irradiation is maximized in the target tissue without delivering harmful doses to surrounding normal tissues.

The present study was conducted to evaluate the potential of $^{211}$At-labelled MAbs as a treatment against solid tumours (> $10^8$ cells) using the intratumour route of injection. Subcutaneous human osteosarcoma xenografts in athymic mice were used as a model and the TP-3 (antigen-specific) and hIgGκ (non-specific) antibodies were used as carrier molecules for $^{211}$At. By the use of an X-ray-counting probe and microautoradiography, tumour clearance and microdistribution of the internal α-particle-emitting source was measured and tumour radiation doses could be estimated. Based upon these results, predictions about decay intensity and microdistribution requirements for clinical tumour treatment with internal α-particle emitters could be made.

### MATERIALS AND METHODS

### Preparation of the radiolabelled compounds

The MAb TP-3 of subclass IgG2b (Bruland et al, 1986), which recognizes an 80-kDa antigen commonly expressed in human osteosarcomas (Bruland et al, 1988), was used as antigen-specific antibody, while polyclonal human IgGκ (Paus and Nustad, 1989) was used as a non-specific antibody.

$^{211}$At was produced at the Scanditronix MC 35 cyclotron at Oslo University by bombarding targets consisting of natural bismuth fused onto aluminium backings with a beam of 28 MeV α-particles. Two hours of cyclotron irradiation using a beam current of 11 μA resulted in approximately 250 MBq $^{211}$At in the target. Astatine was extracted from the target using a dry distillation procedure, which has been previously described (Larsen et al, 1993). Briefly, a still containing the target was heated to 650°C and the volatile astatine was carried by argon to an ice-cooled condensation trap where the astatine was collected in 300 μl of chloroform. Using this procedure, 40–70% of the target activity was recovered after 50 min of distillation. The $^{211}$At was thereafter conjugated to the antibodies as presented in detail (Larsen et al, 1994*a*) The RICs were purified by elution through a Sephadex G-25 PD-10 column (Pharmacia, Sweden) using 0.1 M phosphate-buffered saline (PBS) buffer. The labelling yield was approximately 60% for both proteins.

The antigen binding fractions of [$^{211}$At]TP-3 and non-specific binding of [$^{211}$At]hIgGκ were measured after 1 h of incubation with $10^7$ OHS osteosarcoma cells in 0.5 ml of phosphate-buffered saline (pH 7.4). The cells were washed and counted and the specific bound fraction was determined as described (Larsen et al, 1994*a*).

The antigen binding fraction of the [$^{211}$At]TP-3 measured as binding to OHS cells in suspension ranged from 66% to 81%. The non-specific binding of [$^{211}$At]hIgGκ to OHS cells was less than 4%.

### Tumour model

The OHS xenograft model was used as previously reported (Bruland et al, 1987). Briefly, small pieces of OHS human osteosarcoma xenografts were implanted subcutaneously in the flanks of male athymic Nu/Nu mice. After approximately 2 weeks, when the tumours had become vascularized and had reached an average diameter of 6.6 mm (range 5.5–8.0 mm), groups of animals with similar average tumour size and size distribution were treated with a single intratumour injection of either saline (the control group), [$^{211}$At]TP-3 IgG or [$^{211}$At]hIgGκ. The animals were anaesthetized before injections. All animals were treated in accordance with guidelines on the care of laboratory animals in cancer research (e.g. UFAW, 1987; UKCCCR, 1988).

### Injections of therapeutic preparations

Dilutions of the MAbs were made containing activity dosages of 4, 2 and 1 MBq $g^{-1}$ tumour when a solution (volume adjusted for each tumour individually) corresponding to 10% of the total tumour volume was injected. The control group was injected with a similar volume of isotonic saline. The injections were performed when the specific activity of the radioimmunoconjugates was within 45–55 Mbq $mg^{-1}$ antibody. All of the injections were performed using a 25-μl Hamilton syringe.

### Measurements of $^{211}$At-labelled antibodies in the tumours

Tumour radioactivity was measured in living animals using a hand-held probe (Neoprobe, Columbus, OH, USA) made for the detection of X-rays and soft γ-rays, with the control unit (RIGS model 1000 control unit) set at 2 s counting time. This probe was originally developed for radioguided surgery and is strongly shielded against low-energy X-rays, except for the detection area in the front to reduce the possibility of interference from radioactivity in the body of animals, the probe front was kept perpendicular to the body surface during acquisition of tumour counts. The radioactivity counts in tumour was measured within 1 min after injection and then at various time points up to 24 h. Additionally, the animal body radioactivity count rate was measured by directing the probe at the front of the neck (thyroidal region) and at the abdomen at 12 h after injections. To verify the reliability of the detection method, five animals were sacrificed 20 h after injection and the tumour radioactivity was counted before and after removal of the tumours from the animals. No significant deviation in the tumour counts was observed for the two sets of measurements. The hand-held probe was calibrated against an LKB Wallac 1260 Multigamma II counter (Bromma, Sweden), with window setting for the 77–92 keV X-rays accompanying $^{211}$At decay, and also against a Capintec CRC-7 (Ramsey, NJ, USA) dose calibrator (set for $^{133}$Xe). Calibration was performed with both solutions and with

© Cancer Research Campaign 1998

tumours from sacrificed animals. The overall counting efficiency of the probe was determined by measuring activity in tumours while still implanted in animals as well as after dissection and by comparing these counts with the counts measured in the LKB counter. The fraction of detected counts vs the actual number of $^{211}$At transformations in the tumours was measured to be 3.9% for the Neoprobe system. The difference in the two numbers was due to the abundance of the Po X-rays (44%), the geometry of the detector vs source and the probes' efficiency for the 77–92 keV photons.

### Cumulated activity in the tumours

Based on the retention data the cumulated activity, $\tilde{A}$, in the tumours was calculated according to the linear trapezoid method (e.g. Yuan, 1993). It was assumed that the clearance of radioactivity from the tumours followed two-component kinetics, i.e. a rapidly clearing and a slowly clearing component. Cumulated activity values were determined both for the total activity and the activity from the rapidly clearing component on the curve.

### Microautoradiography

Animals that had received injections of 4 MBq g$^{-1}$ tumour were sacrificed 12 h after injection of the RICs. The tumours were removed and thin slices of the tumour tissue were dried in air and coated with hypercoat emulsion LM-1 (Amersham, UK) for high-resolution microautoradiography. After 3 days, the emulsion-coated slices were developed with Ilford Phenisol liquid and also counterstained with haematoxylin for light microscopy. Tissues of tumours treated both with antigen-positive and antigen-negative RIC was studied and the different areas of the tumour slices showing the α-particle tracks were photographed using a Zeis Ultraphot photo microscope (Zeis, Germany) at × 680 magnification.

### Dose estimates

The radiation considered was the α-particles of $^{211}$At and $^{211}$Po and their recoil nuclei. The absorbed radiation from the X-rays and γ-rays were assumed negligible because of their relative low energy deposition (< 1% of the total energy deposition). The average energy per transition, $\Delta i$, used for the calculations was $1.1 \times 10^{-12}$ Gy kg Bq$^{-1}$ s as adapted from Weber et al (1989). An absorbed fraction, $\phi$, value of 1.0 was assumed because of the large diameters of the tumours compared with the α-particle ranges (about 100:1 ratio).

The radiation doses to the tumours were estimated by considering two different zones of the tumours, i.e. the zones defined by the inner and the outer 50% of the radius. The average radiation dose to each zone was calculated according to the uniform isotropic model (Loevinger et al, 1991) assuming uniform distribution of the radioactivity within each of the tumour zones. Estimates were based on the assumption that the rapidly clearing component was uniformly distributed in the whole tumour, while the slowly clearing component was heterogeneously distributed according to the autoradiograph.

The total radiation dose to the tumour tissue could thereby be expressed in the following equation: $D = D_1 + D_2$, where $D_1$ is the dose from the the rapidly clearing component and $D_2$ is the dose from the slowly clearing component.

The average number of α-particle tracks in the tumours was determined from 20 measurements in the inner half-radius zone and likewise 20 measurements in the outer half-radius zone of each tumour. Each measurement was performed by counting the number of α-particle tracks in an area of approximately $10^4$ μm$^2$ using a phase-contrast microscope (Olympus CH-2, Japan) with a × 400 magnification. The outer half-radius was weighted by a factor of 8 according to the difference in total volume of the two zones. Hence, the average number of α-particle tracks in the whole tumour was estimated according to the following equation: $n = (8n_{ou} + n_{in})/9$, where $n_{ou}$ and $n_{in}$ is the average number determined in the outer and the inner half-radii of the tumours. The $n$ value was considered to be proportional to the cumulated activity, $\tilde{A}$, of the slowly clearing RIC component.

### Measurement of the therapeutic response

The long and short tumour diameters were determined using calliper measurements and the average diameter was calculated. As the tumours were in general close to spherical in shape, the tumour volume was determined using the equation: $V = [(D + d)/2]^3 \times \pi/6$ ($D$, long diameter; $d$, short diameter). The tumour diameters were measured immediately before the injections and at intervals of 2–3 days after the treatment. Animals were followed to day 40 or were sacrificed earlier if the tumour volume increased beyond 2500 mm$^3$.

### Statistical analyses

The tumour growth in the two different groups was compared using the Wilcoxon rank-sum test (e.g. Remington and Schork, 1985) while the student's $t$-test function in the computer program Sigma Plot (Jandel Scientific, CA, USA) was used to compare retention data.

### RESULTS

### Tumour retention of the RICs

In Figure 1A, the relative radioactivity contents in the tumours as followed to approximately 24 h post injection are presented. No significant dependency of injected activity dosage was observed, and the data were therefore pooled for the three dosages of each preparation. The reduction in radioactivity counts is attributed to both the decay of the radionuclide and the clearance of radioimmunoconjugate from the tumour tissue. For comparison, the curve illustrating the physical decay of $^{211}$At was plotted. Approximately 50% of the injected activity left the tumours within 0.5 h after injection for both the specific and non-specific compound. At the earlier time points, the tumour retention was similar for both the specific and the non-specific antibody but, at the 11.0 h point, hIgGκ showed significantly lower relative radioactive counts than TP-3 at the 12.0-h point (Student's independent $t$-test, $P < 0.05$). This was also observed for the comparison of the 23.2-h hIgG point vs 24.1-h TP-3 point. Expressed as per cent of injected activity dosage per gram of tumour the retained activity was 203 ± 93 ([$^{211}$At]TP-3, 24.1 h) and 57 ± 22 ([$^{211}$At]hIgGκ, 23.2 h) at the last time point. Figure 1B presents the decay-corrected retention of the radioimmunoconjugates. The curves show that about 50% of both preparations cleared from the tumours in approximately 0.5 h and after that the clearance was on average 0.81% h$^{-1}$ for [$^{211}$At]TP-3 and 1.9%h$^{-1}$ for [$^{211}$At]hIgGκ.

© Cancer Research Campaign 1998

**Figure 1** Retention of [$^{211}$At]TP-3 (●) and [$^{211}$At]hIgGκ (○) in OHS osteosarcoma xenografts in mice. **A** shows the radioactivity counts at the various time points. The dotted line represents the physical decay curve of $^{211}$At. **B** shows the decay-corrected tumour retention of the two radioimmunoconjugates

In Table 1, the standardized count rates (i.e. per cent of body counts vs tumour counts) at 12 h after injection are presented. The probe was directed at upper abdomen (in front of stomach) and neck (in front of thyroid), which were the locations showing maximum normal tissue counts, probably because these regions show strong accumulation of even minor amounts of dehalogenated nuclide (for unblocked animals) as well as having blood-rich tissues in close vicinity. Significantly higher relative activity values were observed with the non-specific compared with the specific antibody, with on average a 46% higher relative value at the abdomen ($P = 0.045$) and 79% higher relative value at the neck ($P = 0.032$) for all dose groups combined for each antibody.

### α-Particle autoradiographs

The α-particle tracks observed with microautoradiography indicate significant differences in the distribution pattern within the tumour tissue between specific and non-specific RICs. The autoradiographs of [$^{211}$At]TP-3 show a pronounced heterogeneity in the distribution of α-particle tracks (Figure 2A and B), which may be related to the trapping of the antibody by cells near the injection sites. The autoradiographs of the non-specific [$^{211}$At]hIgGk show α-particle tracks distributed more homogeneously in the tumour tissue (Figure 2C and D), although areas with low α-particle track density also occurred for this compound (not shown in the figure). Figure 3 shows the α-particle tracks of [$^{211}$At]TP-3 in a larger region of the tumour at a lower magnification. A distinct border indicating antigen excess is observed.

### Cumulated activity and tumour dose

The cumulated activity, $\tilde{A}$, in the tumours as calculated for an injected activity dosage of 4 MBq/ g$^{-1}$ tumour was for [$^{211}$At]TP-3 (62.0 ± 22.8) × 10$^{9}$ Bq s with (17.2 ± 5.6) × 10$^{8}$ Bq s due to the rapidly clearing component and for [$^{211}$At]hIgGk (39.2 ± 12.0) × 10$^{9}$ Bq s with (17.3 ± 5.2) × 10$^{8}$ Bq s from the rapidly clearing component. The estimated radiation doses to the inner and the outer segments of the tumours are presented in Table 2. A major variation in the calculated doses, which was particularly large for [$^{211}$At]TP-3, is indicated.

### Tumour growth delay

The average tumour growth delay for each treatment group determined as a function of time is presented for [$^{211}$At]TP-3, in Figure 4A and for [$^{211}$At]hIgGk in Figure 4B. The plots indicate a growth delay according to the injected activity dosage. The tumour volume-doubling and -quadrupling times are presented in Figure 5. The volume-doubling and -quadrupling times increased by a factor of 2.1 and 2.3 for [$^{211}$At]TP-3 and 2.1 and 2.0 for [$^{211}$At]hIgGκ, respectively, for the highest activity dosage compared with the control. The 4 MBq g$^{-1}$ [$^{211}$At]TP-3 and both 2 and 4 MBq g$^{-1}$ [$^{211}$At]hIgGκ groups had significantly (Wilcoxon rank-sum; $P < 0.05$) longer volume-doubling times compared with the control group. Except for the 1 MBq g$^{-1}$ activity dosage group of [$^{211}$At]-TP-3, the groups treated with $^{211}$At preparations had a significantly ($P < 0.05$) increased volume-quadrupling time when each group was compared individually with the control group. Isodosage group comparisons of [$^{211}$At]TP-3 and [$^{211}$At]hIgGκ indicated no significant differences in volume-doubling or -quadrupling times. Combined for all three activity dose levels, 5 of 23 tumours were strongly growth inhibited (sizes less than

**Table 1** Radioactive counts measured at the abdomen and the neck 12 h after intratumour injection[a]

| Injected dosage (MBq g$^{-1}$) | [$^{211}$At]TP-3 | | [$^{211}$At]hIgGκ | |
|---|---|---|---|---|
| | Abdomen | Neck | Abdomen | Neck |
| 1.0 | 30.9±26.2 | 14.0±8.8 | 46.2±21.3 | 28.5±21.1 |
| 2.0 | 21.6±8.1 | 8.4±4.1 | 36.4±13.8 | 19.3±9.5 |
| 4.0 | 30.0±13.5 | 17.6±7.0 | 37.3±13.8 | 19.4±10.1 |
| All dosages combined | 27.7±17.7 | 13.4±7.6 | 40.4±16.6 | 22.6±14.6 |

[a]Relative percentage compared with tumour counts.

© Cancer Research Campaign 1998

Figure 2 Microautoradiography of tumour slices showing α-particle tracks made by the decay of $^{211}At$ bound to TP-3 (**A** and **B**) and hIgGκ (**C** and **D**). The photographs show areas of approximately 130 × 170 μm of the tumour centre (**A** and **C**) and the tumour rim (**B** and **D**) and were taken using a photomicroscope at ×680 magnification

1000 $mm^3$ within 40 days) by the [$^{211}$At]TP-3 and 3 of 24 tumours were strongly growth inhibited by the [$^{211}$At]hIgGκ, while none in the control group (0 out of 14) showed a similar reduced growth rate.

### DISCUSSION

To improve radioimmunotherapy as an anti-cancer strategy, the physical half-life of the radionuclide and radiation quality have to be matched carefully for each type of cancer. Local application as opposed to intravenous injection may be a way to improve the pharmacokinetics in targeting of locally confined cancers. Intracavitary or intratumour injections of RICs using nuclides of relatively short half-life (in the range of hours) can ensure a high concentration of the radioactive compound in the target area compared with tissues distant from the injection site (Roeske and Chen, 1993). The use of α-particle emitters can reduce dose-rate

© Cancer Research Campaign 1998 

Figure 3 Microautoradiography showing the border region between high and low density of α-particle tracks for [$^{211}$At]TP-3. The photograph was taken using a photomicroscope at ×100 magnification

Table 2 Estimated doses[a] (Gy) to tumours after intratumour injection of $^{211}$At-radioimmunoconjugates

| Tumour segment | [$^{211}$At]TP-3 | [$^{211}$At]hIgGκ |
|---|---|---|
| Inner half-radius | 276.9 (32.8–747.3) | 44.8 (7.0–90.7) |
| Outer half-radius | 45.2 (2.8–92.4) | 45.6 (7.0–124.3) |

[a]The average dose (range) for an injected activity dosage of 4.0 MBq g$^{-1}$ tumour.

Figure 4 Growth of OHS osteosarcoma xenografts after treatment with a single injection of [$^{211}$At]TP-3 (A) and [$^{211}$At]hIgGκ (B). Group symbols: ●, control; ▽, 1.0 MBq g$^{-1}$; □, 2.0 MBq g$^{-1}$; ○, 4.0 MBq g$^{-1}$

dependency of the radiation treatment and strongly reduce the radioresistance caused by hypoxic tumour cell subpopulations (Hall, 1994). The relatively slow release of radiolabelled immunoglobulins into the blood pool after intratumour injections have been demonstrated previously (Rowlinson-Buza et al, 1991), and it was confirmed in the current study. This route of administration may therefore be particularly interesting with α-particle emitters of short half-life – the short half-life ensuring that a large fraction of the injected radioactivity decays in the tumour before release into the blood stream and the strongly limited radiation range confining the radiation to the tumour tissue cells, sparing surrounding normal tissue.

The main advantage using an antigen-specific antibody, as demonstrated in this study, was that a substantially higher fraction of the injected dose decayed within the tumours with the specific RIC than with the non-specific RIC, as illustrated by, on average, a 58% higher cumulated activity with TP-3 than with hIgGκ. Although the normal tissue distributions for the two RICs were not measured in detail, the difference in radioactivity level as measured at abdomen and neck as well as the difference in tumour retention indicate that reduced normal tissue radiation exposure can be achieved with the antigen-specific compared with the non-specific RIC. As seen in Figure 1, the leakage of the RIC from tumour followed a two-component kinetic, with in excess of 40% of injected activity clearing the tumours within the first 2 h. Possible causes for this leakage may be draining as a result of a combination of high intratumour pressure and capillary damage caused by the injection procedure. Although some dehalogenation has been observed with [$^{211}$At]RIC injected intravenously, this is an unlikely cause for the fraction of rapidly clearing radioactivity from the tumour. Generally, if dehalogenation occurs, it is a relatively slow process for these types of RICs. Previously, we have demonstrated that intravenously injected [$^{211}$At]TP-3 localized stably for up to 48 h in OHS tumour (Larsen et al, 1994*a*), which suggests that intratumour dehalogenation is not a significant problem for the OHS model within this time frame.

The microautoradiographic images used in the radiation dose estimates showed large variations in density of α-particle tracks within each tumour zone studied. The difference in average α-particle track density was relatively large in the two zones compared for [$^{211}$At]TP-3 but not for [$^{211}$At]hIgGκ. As indicated in Table 1, there were large radiation dose ranges within each zone, implying variability in the microdistribution not acknowledged by the uniform isotropic two-zone model. Significant variability in α-particle track density was observed both in the centre and at the edges of the tumours. The average doses to the outer tumour zone were found to be similar for both antibodies, signifying that some diffusion of [$^{211}$At]TP-3 also occurred. This is not likely to be due to saturation, as approximately $3.2 \times 10^{14}$ antibody molecules were injected per gram of tumour at the highest dose level. OHS cells

© Cancer Research Campaign 1998

**Figure 5** Growth delay presented as time for OHS tumours to reach two and four times the initial volume (mean ± s.d.) after treatment with [$^{211}$At]TP-3 (**A**) and [$^{211}$At]hIgGκ (**B**)

have been shown to contain on average 7.4 × $10^5$ antigens per cell in vitro (Larsen et al, 1994*b*), while the OHS tumour line has been found to have up to six times higher antigen expression compared with the cell line (Hjelstuen et al, manuscript in preparation). Assuming $10^9$ cells per gram, the tumour contained in excess of 7 × $10^{14}$ antigens. The approximate 25% of the TP-3 that was not immunoreactive, an unknown fraction of more loosely bound low-affinic TP-3 with monovalent binding capability and also binding of immunoreactive TP-3 to interstitially shedded antigen may account for the [$^{211}$At]TP-3 observed in the outer tumour zone.

For intratumour and regional injections of a α-particle-emitting radiopharmaceutical to be a therapeutically useful strategy, high tumour retention as well as a relatively uniform microdistribution of radiation source among the tumour cells are vital. As demonstrated in this study, a high affinity of the antibody to the tumour cell antigens may not in every sense be advantageous. If the number of available antigens in the tumour tissue is high compared with the number of injected antibody molecules, most binding occurs at the cells near the site of injection. Tumour tissue areas distant to the injection site are thereby only exposed to low concentrations of RIC causing 'hot' and 'cold' spots in the tumour. These problems may be dealt with either by using antibodies with lower antigen affinity or by using competitive inhibition of antigen binding by diluting the RIC with cold antibody (i.e. reduce specific activity of the preparations). Another possible approach previously shown to increase residence time in tumour and decrease the antigen affinity (Delgado et al, 1996) would be to use a polyethylene glycol modification of the antibody used as radionuclide carrier. Alternatively, carrier molecules of lower size causing increased diffusion of the radionuclide within the tumour tissue may secure a more uniform distribution of α-particles. Examples of such molecules are antibody fragments for tumours expressing antigens; for rapidly proliferating tumours, the DNA-incorporated compound [$^{211}$At]astatodeoxyuridine (Vaidyanathan et al, 1996) may be an alternative. As tumour retention is likely to be reduced with smaller molecules, it would be mandatory that the body clearance for these types of molecules would be rapid to keep normal tissue exposure low.

Despite the heterogeneous radiation dose distribution demonstrated in this investigation, the tumour growth delay was dependent on the radioactivity level injected in a dose–response trend. Complete tumour inactivation may therefore be obtained by increasing the injected activity dosage further. The tumour to body weight ratio of approximately 1:130 was high in this study and would correspond to a 0.56-kg tumour in a human with a weight of 75 kg. If intratumour infusions were to be used in humans, it would more likely be in cystic tumours below 100 g. According to an estimate based on (1) a tolerable intravenously injected activity dosage of 10 Mbq $kg^{-1}$ bodyweight in humans, corresponding to approximately 20% of the weight-adjusted maximum-tolerable dosage of [$^{211}$At]astatide in mice (Cobb et al, 1988), and (2) a similar retention of RIC in clinical tumours as observed in our model, a 50-g tumour receiving 1.5 GBq of [$^{211}$At]RIC that retains 50% of the activity (i.e. 750 MBq to the body) would receive an average radiation dose of 600 Gy. Assuming the least exposed areas of the tumour could be exposed to 15% of the average radiation dose (i.e. 90 Gy) or more, the probability for complete tumour inactivation would be high.

In conclusion, in spite of the heterogeneous distribution of the source within the tissue, a significant growth inhibition of human tumour xenografts implanted in mice was observed after intratumour injections of radioimmunoconjugates emitting α-particle radiation. The use of radionuclides with relatively short half-lives ensures high tumour radiation doses with limited normal tissue exposure from the radioactivity cleared from the tumour. A successful treatment using this method depends upon both tumour retention and microdistribution of the radiation. Heterogeneous radiation dose distribution is likely to occur in larger tumours, and this may be one of the main disadvantages of α-particle emitters in molecular-targeted radiotherapy. Non-uniformity in the deposition of radiation can be acceptable only if all tumour cells are exposed to a threshold radiation dose sufficient for clonogenic inactivation.

*© Cancer Research Campaign 1998*

### ACKNOWLEDGEMENTS

The authors wish to thank Eivind Olsen, Department of Physics, University of Oslo, Norway, for performing the cyclotron irradiations, Ellen Kilde Evensen and Marita Martinsen for their assistance with the animal experiments and Susan L. Reeves, Pathology Photography Laboratory, Duke University Medical Center for preparing the photographs. This study was financially supported by the Norwegian Cancer Society grants 90077 and C 96070.

### REFERENCES

Brown I (1986) Astatine-211: its application in cancer therapy. *Appl Radiat Isot* **37**: 789–798

Bruland ØS, Fodstad Ø, Funderud S and Pihl A (1986) New monoclonal antibodies specific for human sarcomas. *Int J Cancer* **37**: 27–31

Bruland ØS, Fodstad Ø and Pihl A (1987) Selective localization of two radiolabelled anti-sarcoma monoclonal antibodies in human osteosarcoma xenografts. *Br J Cancer* **56**: 21–25

Bruland ØS, Fodstad Ø, Stenwig A and Pihl A (1988) Expression of a novel human osteosarcoma associated cell surface antigen. *Cancer Res* **48**: 5302–5309

Bruland ØS, Skretting A, Solheim ØP and Aas M (1996) Targeted radiotherapy of osteosarcoma using $^{153}$Sm-EDTMP. *Acta Oncol* **35**: 381–384

Cobb LM, Harrison A and Butler SA (1988) Toxicity of astatine-211 in the mouse. *Human Toxicol* **7**: 529–534

Delgado C, Pedley C, Herraez A, Boden R, Boden JA, Keep PA, Chester KA, Fisher D, Begent RHJ and Francis GE (1996) Enhanced tumour specificity of an anti-carcinoembryonic antigen Fab′ fragment by poly(ethylene glycol) (PEG) modification. *Br J Cancer* **73**: 175–182

Hadley RW, Wilbur DS, Gray MA and Archer RW (1991) Astatine labelling of an antimelanoma antibody and its Fab fragment using N-succinimidyl p-astatobenzoate: comparison in vivo with the p-[$^{125}$I]iodobenzoyl conjugate. *Bioconjug Chem* **2**: 171–179

Hall EJ (1994) *Radiobiology for the Radiologist*. 4th edn. JB Lippincott: Philadelphia

Harrison A and Royle L (1984) Preparation of a $^{211}$At-IgG conjugate which is stable in vivo. *Int J Appl Radiat Isot* **35**: 1005–1008

Harrison A and Royle L (1987) Efficacy of astatine-211 labelled monoclonal antibody in treatment of murine T-cell lymphoma. *NCI Monogr* **3**: 157–158

Humm JL (1987) A microdosimetric model of astatine-211 labelled antibodies for radioimmunotherapy. *Int J Radiat Oncol Biol Phys* **13**: 1767–1773

Huneke RB, Pippin CG, Squire RA, Brechbiel MW, Ganzow OA and Strand M (1992) Effective α-particle-mediated radioimmunotherapy of murine leukemia. *Cancer Res* **52**: 5818–5820.

Jardine JL (1975) Decays of $^{211}$At, $^{211}$Po, and $^{207}$Bi. *Phys Rev* **11**: 1385–1391

Kassis AI, Harris CR, Adelstein SJ, Ruth TJ, Lambrecht R and Wolf AP (1986). The in vitro radiobiology of astatine-211 decay. *Radiat Res* **105**: 27–36

Kozak RW, Atcher RW, Ganzow OA, Friedman AM, Hines J and Waldman TA (1986) Bismuth-212-labelled anti-Tac monoclonal antibody: α-particle-emitting radionuclide as modalities for radioimmunotherapy. *Proc Natl Acad Sci USA* **83**: 474–478

Larsen RH and Bruland ØS (1995) Radiolysis of radioimmunoconjugates. Reduction in antigen binding ability by α-particle radiation. *J Labelled Compound Radiopharmaceut* **36**: 1009–1018

Larsen RH, Hassfjell SP, Hoff P, Alstad J, Olsen E, Vergote IB, de Vos LN, Bjørgum J and Nustad K (1993) $^{211}$At-labelling of polymer-particles for radiotherapy: synthesis, purification and stability. *J Labelled Compound Radiopharmaceut* **33**: 977–986

Larsen RH, Hoff P, Alstad J and Bruland ØS (1994*a*) Preparation and quality control of $^{211}$At-labelled and $^{125}$I-labelled monoclonal antibodies. Biodistribution in mice carrying human osteosarcoma xenografts. *J Labelled Compound Radiopharmaceut* **34**: 773–785

Larsen RH, Bruland ØS, Hoff P, Alstad J, Lindmo T and Rofstad EK (1994*b*) Inactivation of human osteosarcoma cells in vitro by $^{211}$At-TP-3 monoclonal antibody: Comparison with astatine-211-labelled bovine serum albumin, free astatine-211 and external beam X-rays. *Radiat Res* **139**: 178–184

Larsen RH, Bruland ØS, Hoff P, Alstad J and Rofstad EK (1994*c*) Analyses of the therapeutic gain in the treatment of human osteosarcoma microcolonies in vitro with $^{211}$At-labelled monoclonal antibody. *Br J Cancer* **69**: 1000–1005

Larsen RH, Hoff P, Vergote IB, Bruland ØS, Aas M, de Vos L and Nustad K (1995) α-particle radiotherapy with $^{211}$At-labelled monodisperse polymer particles, $^{211}$At-labelled IgG proteins, and free $^{211}$At in a murine intraperitoneal tumour model. *Gynecol Oncol* **57**: 9–14

Larsen RH, Wieland B and Zalutsky MR (1996) Evaluation of an internal cyclotron target for the production of astatine-211 via the $^{209}$Bi (α, 2n)$^{211}$At reaction. *Appl Radiat Isot* **47**: 135–143

Link EM and Carpenter RN (1992) $^{211}$At-methylene blue for targeted radiotherapy of human melanoma xenografts. *Cancer Res* **52**: 4385–4390

Loevinger R, Budinger TF and Watson EE (1991) *MIRD Primer for Absorbed Dose Calculation* (revised edition). The Society of Nuclear Medicine: New York

Maclis RM, Kaplan WD, Ferrera, JLM, Archer RW, Hines JJ, Burakoff SJ and Coleman CN (1989) Resident Essay Award: alpha particle radio-immunotherapy: animal models and clinical prospects. *Int J Radiat Oncol Biol Phys* **16**: 1377–1387

Paus E and Nustad K (1989) Immunoradiometric assay for αγ- and γγ-enolase (neuron-specific enolase), with use of monoclonal antibodies and magnetizable particles. *Clin Chem* **35**: 2034–2038

Remington RD and Schork MA (1985) *Statistics with Applications to the Biological and Health Sciences*, 2nd edn. Prentice-Hall: Englewood Cliffs, New Jersey

Riva P, Arista A, Vittorio T, Sturiale C, Franchesci G, Spinelli A, Riva N, Casi M, Moscatelli G and Frattarelli M (1994) Intralesional radioimmunotherapy of malignant gliomas. *Cancer* **73**: 1076–1082

Roeske JC and Chen TY (1993) Dosimetry of administered radiolabelled antibodies. *Med Phys* **20**: 593–600

Rowlinson-Busza G, Bamias A, Krausz T and Epenetos AA (1991) Uptake and distribution of specific and control monoclonal antibodies in subcutaneous xenografts following intratumor injection. *Cancer Res* **51**: 3251–3256

Royal Society/Universities Federation for Animal Welfare (UFAW) (1987) Guidelines on the care of laboratory animals and their use for scientific purposes 1. Housing and care. UFAW: London

Sgouros G (1995) Radioimmunotherapy of micrometastases: sidestepping the solid-tumor hurdle (editorial). *J Nucl Med* **36**: 1910–1912

United Kingdom Coordinating Committee on Cancer Research (UKCCCR) (1988) UKCCCR guidelines for the welfare of animals in experimental neoplasia. *Br J Cancer* **58**: 156–160

Vaidyanathan G, Larsen RH and Zalutsky MR (1996) 5-[$^{211}$At]astatodeoxyuridine, an α-particle emitting endoradiotherapeutic agent undergoing DNA incorporation. *Cancer Res* **56**: 1204–1209

Vaughan ATM, Bateman W and Cowan J (1981) The preparation and cytotoxic properties of $^{211}$At-labeled concavalin A bound to cell membranes. *J Radioanal Chem* **64**: 33–39

Vergote IB, Larsen RH, de Vos L, Winderem M, Bjørgum J, Hoff P, Aas M, Tropè C G and Nustad K (1992) Distribution of intraperitoneally injected microspheres labelled with the α-emitter Astatine ($^{211}$At) compared with Phosphorous ($^{32}$P) and Yttrium ($^{90}$Y) colloids in mice. *Gynecol Oncol* **47**: 358–365

Weber DA, Eckereman KF, Dillman LT and Ryman JC (1989) *MIRD: Radionuclide Data and Decay Schemes*. The Society of Nuclear Medicine: New York

Wheldon TE and O'Donnoghue JA (1990) The radiobiology of targeted radiotherapy. *Int J Radiat Biol* **58**: 1–21

Yuan J (1993) Estimation of variance for AUC in animal studies. *J Pharm Sci* **82**: 761–763

Zalutsky MR, Garg PK, Friedman HS and Bigner DD (1989) Labeling monoclonal antibodies and F(ab′)$_2$ fragments with the α-particle-emitting nuclide astatine-211: preservation of immunoreactivity and in vivo localizing capacity. *Proc Natl Acad Sci USA* **86**: 7149–7153

*© Cancer Research Campaign 1998*